# Case report: hepatitis in a child infected with SARS-CoV-2 presenting toll-like receptor 7 Gln11Leu single nucleotide polymorphism

**DOI:** 10.1186/s12985-021-01656-3

**Published:** 2021-09-05

**Authors:** Natália Lima Pessoa, Aline Almeida Bentes, Andrea Lucchesi de Carvalho, Thaís Bárbara de Souza Silva, Pedro Augusto Alves, Erik Vinicius de Sousa Reis, Tayse Andrade Rodrigues, Erna Geessien Kroon, Marco Antônio Campos

**Affiliations:** 1 Laboratório de Vírus, Departamento de Microbiologia, Instituto de Ciências Biológicas, Universidade Federal de Minas Gerais, Belo Horizonte, MG Brazil; 2 Imunologia de Doenças Virais, Instituto René Rachou, Fundação Oswaldo Cruz, Belo Horizonte, MG Brazil; 3 Faculdade de Medicina, Universidade Federal de Minas Gerais, Belo Horizonte, MG Brazil; 4 Hospital João Paulo II, Fundação Hospitalar do Estado de Minas Gerais, Belo Horizonte, MG Brazil; 5 Quibasa Química Básica, Belo Horizonte, MG Brazil

**Keywords:** SARS-CoV-2, Covid-19, Hepatitis, Single nucleotide polymorphism, TLR-7, Children, Innate immune defense, rs 179008, Toll-like receptor 7 Gln11Leu

## Abstract

**Background:**

Covid-19 has the respiratory tract as the main target of infection, and patients present mainly dyspnea, pneumonia, dry cough, and fever. Nevertheless, organs outside the respiratory tract had been reported in recent studies, including the gastrointestinal tract and liver. The host innate immune system recognizes pathogen-associated molecular patterns (PAMPs) through their pattern recognition receptor (PRRs). Toll-like receptor 7 (TLR-7) is a pattern recognition receptor recognizing ssRNA (SARS-CoV-2 is an ssRNA). Polymorphisms are characterized by two or more alternative forms of a distinct phenotype in the same population. Polymorphisms in *tlrs* genes can negatively influence the immune response to infectious diseases. There are several references in the literature to non-synonymous single nucleotide (rs) polymorphisms related to several genes. Some of them are important for the innate immunity, as rs 179008 (*tlr-7*), rs3775291 (*tlr3*), rs8177374 (*tir domain-containing adaptor protein, tirap*), rs1024611 (*monocyte chemoattractant protein-1, mcp-1*) and rs61942233 (*2*′*-5*′*-oligoadenylate synthase-3, oas-3*).

**Case presentation:**

We identified a 5-year-old-male child with gastrointestinal symptoms and fever presenting acholic stool and jaundice, who was positive for SARS-CoV-2 IgM, IgA, and IgG and presenting the Gln11Leu rs 179008 in *tlr-7*. The child presented high levels of aspartate aminotransferase, alanine aminotransferase, bilirubin, C-reactive protein, D-dimer, gamma-glutamyl transferase, alkaline phosphatase, and was negative for serological tests for hepatitis A, B, C, E, HIV 1 and 2, herpes virus, cytomegalovirus, Epstein–Barr virus, and negative for RTqPCR for Influenza A and B, RSV and SARS-CoV-2. We also investigated other SNPs in the *tlr-3* (rs3775291)*, tirap* (rs8177374)*, mcp-1* (rs1024611)*,* and *oas-3* (rs61942233) genes, and no mutation was detected**.** After an interview with the child's caregivers, any possible accidental ingestion of drugs or hepatotoxic substances was ruled out.

**Conclusion:**

To our knowledge, this is the first report of a SARS-CoV-2 caused hepatitis in a male child that has the *tlr-7* Gln11Leu rs 179008, which could impair an efficient initial immune response. The knowledge of the patient's immune deficiency could improve the treatment to correct this deficiency with specific medications.

## Background

Coronavirus disease 2019 (COVID-19) is a significant global public health problem. The severe acute respiratory syndrome coronavirus type 2 (SARS-CoV-2), a single-stranded RNA (ssRNA) virus, has the respiratory tract as the main target of infection, and patients present mainly dyspnea, pneumonia, dry cough, and fever [1]. Nevertheless, the involvement of organs outside the respiratory tract had been reported in recent studies, including the gastrointestinal tract and liver [2–4]. Liver damage of varying degrees was present in 58–78% of patients [3].

The host immune response to SARS-CoV-2 infection plays an important role in the severity of the disease [5]. It is responsible for recognizing pathogen-associated molecular patterns (PAMPs) through their pattern recognition receptor (PRRs). Toll-like receptor 7 (TLR-7) is a PRR that recognizes ssRNA [6]. Polymorphisms are characterized by two or more alternative forms of a distinct phenotype in the same population, and polymorphisms in TLRs can negatively influence the immune response to infectious diseases [7]. There are several references in the literature to non-synonymous single nucleotide (rs) polymorphisms related to several genes (https://www.genecards.org/). Some of them are important for the innate immunity, as rs179008 (*tlr-7*), rs3775291 (*tlr3*), rs8177374 (*tir domain-containing adaptor protein, tirap*), rs1024611 (*monocyte chemoattractant protein-1, mcp-1*) and rs61942233 (*2′-5′-oligoadenylate synthase-3, oas-3*) (https://www.genecards.org/).

Unique loss-of-function variants in X chromosomal *tlr-7* were identified in four young men with severe COVID-19 [8], and rs179008 in *tlr-7* gene have been related with increased risk to progress to advanced liver disease in hepatitis C virus (HCV) infection [9]. In the present case report, we identified a male child with the *tlr-7* gene Gln11Leu single nucleotide polymorphism (rs 179008) with hepatitis and positive serological SARS-CoV-2 test. We also investigated other SNPs in the *tlr-3, tirap, MCP-1,* and *oas-3* genes, and no mutation was detected.

## Case presentation

A 5-years-old male child started with fever, odynophagia, diarrhea, abdominal pain, and vomiting on August 19, 2020. On the 23rd, still with fever, he developed acholic stool and jaundice. The child was hospitalized after medical evaluation and tests that detected hepatitis. The patient has asthma and extensively used beclomethasone spray at a low dose of 100 mcg/day. He had no other diseases, and this was his first hospital stay. On the child's vaccination card, there was one dose of hepatitis A and three doses of hepatitis B. After an interview with the child's caregivers, any possible accidental ingestion of drugs or hepatotoxic substances was also ruled out.

The child remained hospitalized for supportive treatment and tests for 4 days, being discharged with improvement in fever, vomiting, and abdominal pain. However, the child was admitted again 4 days later because he had fever, tiredness, and edema in the lower limbs. He was discharged after 3 days. There was no bleeding or hemodynamic failure at any time. The patient received only supportive treatment.

To investigate the possible cause of this hepatitis in this patient, serological tests for viral hepatitis (hepatitis A, B, C, E, HIV-1 and -2, EBV, and CMV), and tests to evaluate possible autoimmune hepatitis, Wilson's disease, and alpha1-antitrypsin deficiency, were performed (Table 1). All these tests were negative. The child shows a positive IgG for EBV and CMV. As the boy had one positive epidemiology for Covid-19 associated with fever, odynophagia and changes in inflammatory markers, coagulation profile, and D-dimer, a serological test was requested and was positive for IgM, IgG, and IgA for SARS-CoV-2, with the kits Biolisa-CoV-2 IgM, Biolisa-CoV-2 IgG, and Biolisa-CoV2 IgA (Bioclin, Quibasa, Brazil), respectively. The RT-qPCR for SARS-CoV-2 was performed from the nasopharynx and oropharynx 6 days after symptoms and was negative (Table 1). An echocardiogram was performed due to the possibility of multisystem inflammatory syndrome associated with covid-19, but the exam did not show changes in cardiac function or coronary dilation. After hospital discharge, the patient continues to be followed up on an outpatient clinic with a pediatric gastroenterology team, but without other clinical manifestations. The patient underwent cholangioresonance examination in January 2021, but the examination did not show any changes. At this time, the antinuclear antibody was also not reactive. Other diagnostic tests performed for differential diagnoses such as Wilson's disease, alpha-1-antitrypsin deficiency, autoimmune hepatitis, and primary sclerosing cholangitis were negative.

**Table 1:** Laboratory results, hepatitis tests, serological tests, and single nucleotide polymorphism genotype and phenotype

Tests (units)/dates	08/24–26	09/02	11/18	Reference range
Hemoglobin (g/dL)	12.8	10.7	12.9	11.5–13.5
Leukocytes (cells/mm^3^)	10,030	8450	6170	5000–14,500
Platelets (cells/mm^3^)	369,000	589,000	432,000	150,000–400,000
C-Reactive protein (mg/L)	**233**	62	–	< 12
Aspartate aminotransferase (IU/L)	**326**	48	33	10–47
Alanine aminotransferase (IU/L)	**234**	49	19	24–49
Bilirubin/direct bilirubin (mg/dL)	**4.4/4.0**	0.9/**0.6**	0/0	≤ 1.2/≤ 0.4
Gamma-glutamyl transferase (IU/L)	**833**	**239**	22	< 30
Alkaline phosphatase (IU/L)	**770**	329	253	142–335
Albumin (g/dL)	3.9	3.9	4.3	2.9–4.7
Activated partial thromboplastin time (s)	**69**	**62**	**77**	25–35
International normalized ratio (INR)	**1.28**	**1.38**	**1.3**	0.87–1.2
D-dimer (mcg/mL)	**1.46**	**1.83**	–	≤ 0.5
α1-Antitrypsin (mg/L)	**293**	–	–	78–200
Ceruloplasmin (mg/dL)	36	–	–	20–60
Antinuclear antibody	**1:80**	–	–	NR
Anti-smooth muscle	NR	–	–	NR
Seric copper (mcg/dL)	177	–	–	90–190
Urinary copper (mcg/24 hs)	11	–	–	≤ 60
Anti-liver kidney microsome type 1	–	NR	–	NR
SARS-CoV-2 IgG/IgA/IgM	**P/P/P**	–	–	N
VDRL	N	–	–	N
RTqPCR influenza A, B; RSV and SARS-CoV2	N/N/N	–	–	N/N/N
Anti-human immunodeficiency virus 1,2	N	–	–	N
Anti-hepatitis A virus/C virus	N/N	–	–	N/N
Anti-hepatitis E IgM	N	–	–	N
Hepatitis B, HBsAg, anti-HBc-IgM	N/N/N	–	–	N/N/N
Anti-human herpes virus IgM	N	–	–	N
Anti-cytomegalovirus IgG/IgM	P/N	–	–	N
Anti-Epstein–Barr virus IgG/IgM	P/N	–	–	N
TLR-7 T/T (A/A)	–	–	**M**	–
TLR-3 C/C (C/C)	–	–	NH	-
TIRAP C/C (C/C)	–	–	NH	–
MCP-1 A/A (A/A)	–	–	NH	–
OAS-3 C/C (C/C)	–	–	NH	–

Bold means alterations in tests

*MCP-1* monocyte chemoattractant protein 1, *M* mutated, non-functional, *NH* normal homozygote, *OAS-3* 2′-5′-oligoadenylate synthetase 3, *SARS-CoV-2* severe acute respiratory syndrome coronavirus type 2, *SNP* single nucleotide polymorphism, *RSV* respiratory syncytial virus**,** *TIRAP* toll-interleukin 1 receptor domain-containing adapter protein, *TLR-3* toll like receptor 3, *TLR-7* toll like receptor 7, *VDRL* Venereal Disease Research Laboratory, *P* positive, *N* negative, *NR* non-reactive, **–** not done

SNPs were selected based on previously reported associations with higher susceptibility of the host to other viral infections and with *tlr* genes, and proteins from the TLR activated cascade. Thereby, SNP rs179008 in the *tlr-7* gene, rs3775291 in the *tlr-3* gene, rs8177374 in the *tirap* gene, rs1024611 in the *mcp-1* gene, and rs61942233 in the *oas-3* gene were tested. The primers used were: 5*′*-AGAGAGGCAGCAAATGGGAA-3*′* and 5*′*-TAGGAAACCATCTAGCCCCA-3′ for *tlr-7*, 5*′*-GCGAACTTTGACAAATGAAACA -3*′* and 5*′*-CCCAACCAAGAGAAAGCATC-3*′* for *tlr-3*, 5*′*-GGTGCAAGTACCAGATGCT-3*′* and 5*′*-CAACGCATGACAGCTTCTTT-3*′* for *tirap*, 5*′*-CTTCTCTCACGCCAGCAC-3*′* and 5*′*-ACAGTAAACACAGGGAAGGT-3*′* for *mcp-1* and 5*′*-GCTGCTTCAGCCAGTTCA-3*′* and 5*′*-GTCAGTGAGAAGCTCAGCA-3*′* for *oas-3*. To detect SNPs, genomic DNA was extracted from peripheral blood, amplified by polymerase chain reaction (PCR), and sequenced. The genotype was confirmed by aligning the resulted sequence with the reference sequences from GenBank in the software Sequence Scanner 2.0 (Applied Biosystems), and novoSNP (Department of Molecular Genetics—VIB and University of Antwerp).

No base change was detected on rs3775291 (*tlr-3*), rs8177374 (TIRAP), rs1024611 (*mcp-1*), and rs61942233 (*oas-3*), being a normal homozygote for these genes. A base change (A > T) was detected on rs179008 (*tlr-7*) SNP, being a mutated SNP, which causes an amino acid change (Q to L).

The procedures were in accord with the ethical standards of the responsible committee on human experimentation from Instituto René Rachou, Fundação Oswaldo Cruz [CAAE 37207920.6.0000.5091] and with the Helsinki Declaration (1964, amended most recently in 2008) of the World Medical Association and the patient's responsible written consent was obtained.

## Discussion

At the beginning of the Covid-19 pandemic, few infections and severe cases in young adults and almost none in children were reported [1]. On February 8, 2021, Lachassinne et al. reported that the incidence of SARS-CoV-2 infection in children was still low [10]. We wondered why some of the children could be infected and developed a severe Covid-19. Liu and Hill [5] reported that primary immunodeficient patients could be more susceptible to severe infections, posing a high risk to Covid-19. Deficiency in antiviral innate immune signaling (TLRs, TIRAP) [5] or chemokines (MCP-1) essential to call the appropriated immune defense cells or in interferon-induced molecules (OAS-3) would be some targets to find the answer to this question. Initially, we addressed our efforts to verify if there were non-synonymous SNPs in DNA that codify these proteins, using the blood of children who needed to be admitted to the hospital.

Although Covid-19 involves mainly the respiratory tract, there are some representative numbers of articles in the literature showing that several organs, including the liver and gastrointestinal tract, are also affected by SARS-CoV-2 [2–4].

Here we reported hepatitis, with gastrointestinal symptoms and fever in a 5-year-old male child, who was negative for hepatitis A, B, C, E, cytomegalovirus, Epstein Barr, syphilis, HIV, herpes, influenza, and respiratory syncytial virus. Additionally, the boy was positive for SARS-CoV-2 IgM, IgA, and IgG and presented one rs179008 in *tlr-7*. However, although immune tests were positive against SARS-CoV-2, a nasal swab collected 6 days after initial symptoms was RTq-PCR negative. Lack of detection of viral RNA 6 days after the first symptoms could be due to partial elimination of the virus confirmed by the presence of antibodies, or even the collection having been made traditionally only in the nasopharynx and oropharynx, since the child had no respiratory manifestations. Wang et al. 2021 [11] tested different clinical samples and detected SARS-CoV-2 in blood and feces. Kucirka et al. 2020 [12] also raised the question about the predictive value of the RTqPCR, which varies with time from exposure and symptom onset, predicting a reduction of 67% in the positivity on the fourth day.

Hepatitis provoked by SARS-CoV-2 could be associated with the impaired innate immunity against the virus caused by the polymorphism in *tlr-7* rs179008. The same SNP was reported by Fakhir et al. as being one of the causes of the impaired immune response during HCV infection [9]. Azar et al. (2020) reported patients infected with HIV-1, which presented the rs179008, produced lower quantities of TLR-7, resulting in lower production of IFN-1, with consequent higher viral load [13].

## Conclusions

To our knowledge, this is the first report of a SARS-CoV-2 caused hepatitis in a male child that has the rs179008 *tlr-7*, which could impair an efficient initial immune response. The knowledge of the patient's immune deficiency could improve the treatment to correct this deficiency with specific medications.

## Data Availability

All data and information are available without restriction and included in the manuscript.

## Abbreviations

MCP-1: Monocyte chemoattractant protein-1
OAS-3: 2*′*-5*′*-Oligoadenylate synthase-3
PAMPS: Pathogen-associated molecular patterns
PRR: Pattern recognition receptor
SNP: Single nucleotide polymorphism
TLR: Toll-like receptor
Tirap: Tir domain-containing adaptor protein
